# Predicting MCI progression with FDG-PET and cognitive scores: a longitudinal study

**DOI:** 10.1186/s12883-020-01728-x

**Published:** 2020-04-21

**Authors:** Lirong Teng, Yongchao Li, Yu Zhao, Tao Hu, Zhe Zhang, Zhijun Yao, Bin Hu, Alzheimer’ s Disease Neuroimaging Initiative (ADNI)

**Affiliations:** 1 Department of Obstetrics and Gynecology, Peking Union Medical College, Chinese Academy of Medical Sciences, Beijing, 100032 P.R. China; 2 Key Laboratory of Wearable Computing of Gansu Province, Lanzhou University, Lanzhou, 730000 P.R. China

**Keywords:** FDG-PET, Mild cognitive impairment, Dynamic features, Cognitive scores, Classification

## Abstract

**Background:**

Mild cognitive impairment (MCI) is an intermediate stage between normal aging and dementia. Studies on MCI progression are important for Alzheimer’s disease (AD) prevention. 18F fluoro-deoxy-glucose positron emission tomography (FDG-PET) has been proven to be a powerful tool for measuring cerebral glucose metabolism. In this study, we proposed a classification framework for MCI prediction with both baseline and multiple follow-up FDG-PET scans as well as cognitive scores of 33 progressive MCI (pMCI) patients and 46 stable MCI (sMCI) patients from the Alzheimer’s Disease Neuroimaging Initiative (ADNI).

**Method:**

First, PET images were normalized using the Yakushev normalization procedure and registered to the Brainnetome Atlas (BNA). The average metabolic intensities of brain regions were defined as static features. Dynamic features were the intensity variation between baseline and the other three time points and change ratios with the intensity obtained at baseline considered as reference. Mini-mental State Examination (MMSE) scores and Alzheimer’s disease Assessment Scale-Cognitive section (ADAS-cog) scores of each time point were collected as cognitive features. And F-score was applied for feature selection. Finally, support vector machine (SVM) with radial basis function (RBF) kernel was used for the three above features.

**Results:**

Dynamic features showed the best classification performance in accuracy of 88.61% than static features (accuracy of 78.48%). And the combination of cognitive features and dynamic features improved the classification performance in specificity of 95.65% and Area Under Curve (AUC) of 0.9308.

**Conclusion:**

Our results reported that dynamic features are more representative in longitudinal research for MCI prediction work. And dynamic features and cognitive scores complementarily enhance the classification performance in specificity and AUC. These findings may predict the disease course and clinical changes in individuals with mild cognitive impairment.

## Background

Alzheimer’s disease (AD), the most common form of dementia, is a progressive, irreversible and currently incurable neurodegenerative disease [1]. With the increasing of aging population, the morbidity rate of AD has significantly increased [2]. Previous studies reported that more than 26.6 million people suffered from AD in 2006 and 1 in 85 individuals would be affected by 2050 [3]. Mild cognitive impairment (MCI) is considered a transition stage between normal aging and AD, and conversion of patients with MCI occurs at an annualized rate of 10 to 15% [4, 5]. Therefore, it is vital to predict if the conditions of MCI patients would deteriorate and lead to AD within a few years, or remain stable for a long duration [6].

Neuroimaging is a powerful tool for monitoring disease progression in dementing illness [7]. Multiple studies focused on structural atrophy [8], pathological amyloid deposition [9] and metabolic alteration [10, 11] to identify efficient features that can detect AD and MCI. In the past decades, FDG-PET which measures cerebral glucose metabolism, has been reported as an impactful MCI biomarker [12–15]. Mosconi et al. found that hypo-metabolism was indicative of potential MCI progression in the inferior parietal cortex [14]. Decreased FDG uptake was reported in posterior cingulate, temporoparietal, and prefrontal association cortex of patients with probable AD by Herholz [12]. Meanwhile, Chetelat et al. found converters had lower uptake in the right temporoparietal cortex compared with non-converters [13]. It was also reported by Ossenkoppele et.al that FDG uptake was reduced at follow-up in the AD group in frontal, parietal and lateral temporal lobes [15]. In addition to FDG-PET, other modalities have been used, including magnetic resonance imaging (MRI) [16–20], and cerebrospinal fluid (CSF) [21–24]. Previous studies have implemented metabolic intensity of FDG-PET images as features, which achieved 85.1% accuracy in classifying pMCI from sMCI at the conversion time [25] and 72.5% accuracy when combining features in baseline and 12 months follow-up [26]. M. Pagani achieved sensitivity of 92% and specificity of 91% in discriminating MCI from healthy controls when implementing metabolic differences from FDG-PET as dynamic features [27]. However, the classification performance of MCI patients needs to be improved by constructing effective classification framework.

Two study types have been applied to assess AD and MCI, including cross-sectional and longitudinal designs. In cross-sectional studies, data for only one time point is involved (i.e., the first screening data) [21, 28–30]. At baseline, the number of subjects at different stages (i.e. AD, NC and different kind of MCI) and that of different modalities (i.e., MRI, PET, fMRI etc.) are complete. Due to greater data availability at baseline, cross-sectional data might benefit from higher statistical power. In longitudinal studies, data for multiple time points are collected, which may provide complementary information to single time point [18, 19, 31–34]. Indeed, longitudinal data can reflect the variation trend, both in structure [18, 19] and cognitive of individual features [32] contrasting with single time point. As MCI is a disease evolving over time, longitudinal data may have a great impact on its classification and detection for some lesions in the brain.

**Fig. 1:**
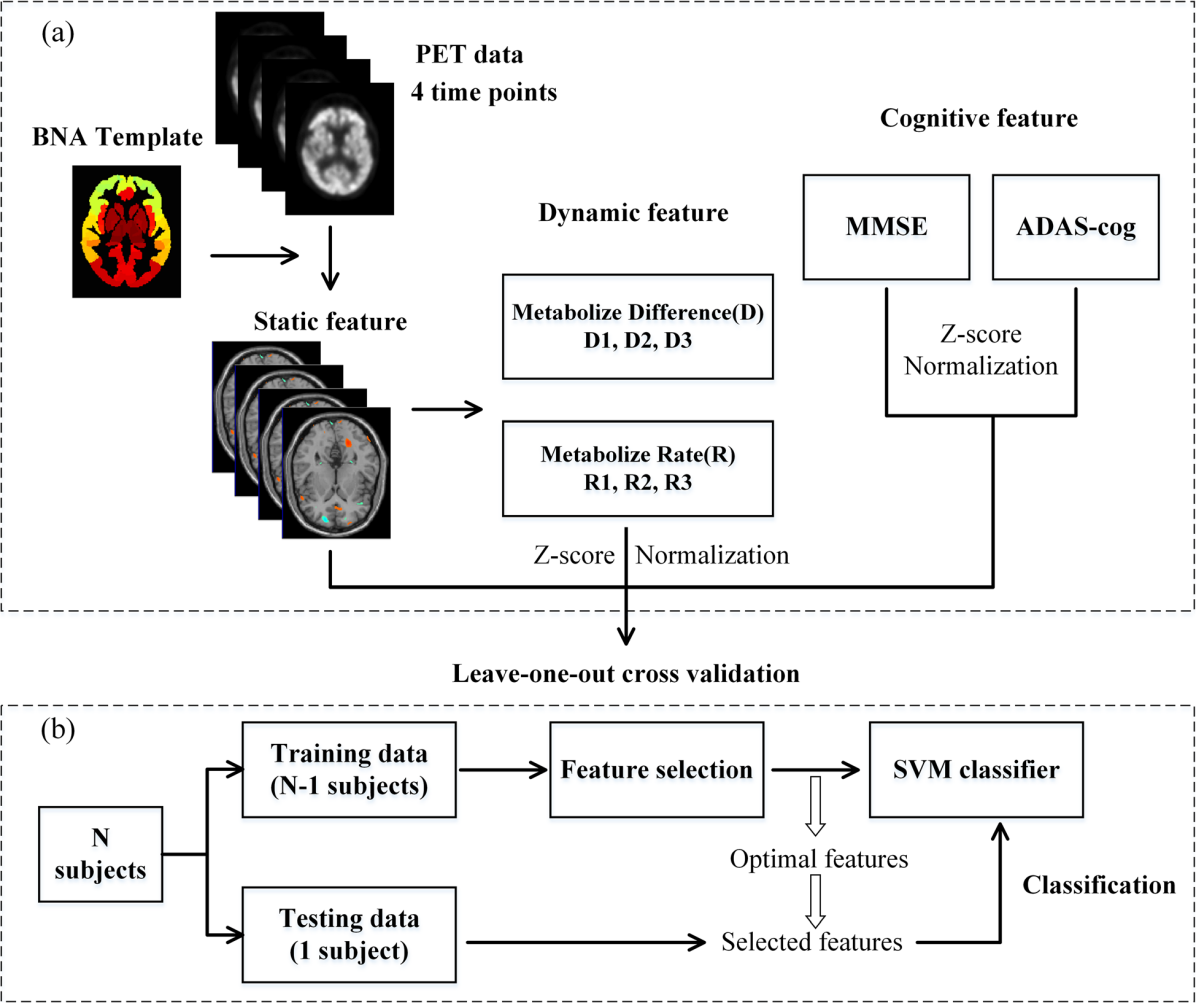
The classification framework. Static feature, dynamic feature, and cognitive feature extraction (**a**). The LOO cross-validation classification evaluation process (**b**)

In this study, we attempted to identify progressive MCI (pMCI) and stable MCI (sMCI) using longitudinal FDG-PET data. We first parceled FDG-PET images of each time point into 246 brain regions [35], whose average metabolic intensities were considered static features. Two types of dynamic features were defined, including intensity difference (D) between baseline and the other three time points and change ratio (R) using the intensity at baseline as reference. Cognitive features were the MMSE scores and ADAS-cog scores of 4 time points. Then all the feature vectors were stacked to form a feature matrix. F-score was used for feature selection. Finally, leave-one-out (LOO) cross-validation was performed for classification with support vector machine (SVM). The classification framework is shown in Fig. 1.

## Methods

### Alzheimer’s disease neuroimaging initiative

Data used in this article were obtained from the ADNI database (http://adni.loni.ucla.edu). ADNI was launched in 2003 by the National Institute on Aging, the National Institute of Biomedical Imaging and Bioengineering (NIBIB), the Food and Drug Administration (FDA), private pharmaceutical companies, and non-profit organizations, as a $60 million,5-years public-private partnership. The subjects have been recruited from over 50 sites across the U.S. and Canada. The primary goal of ADNI is to test whether serial magnetic resonance imaging (MRI), positron emission tomography (PET), other biological markers, and clinical and neuropsychological assessment can be combined to measure the progression of MCI and AD. For up-to-date information, visit www.adni-info.org.

### Subjects

The general inclusion criteria for MCI are: MMSE score between 24 and 30 (inclusive) and a Clinical Dementia Rating (CDR) of 0.5; memory complaint; no significant levels of impairment in other cognitive domains; essential preservation of daily living activities, and absence of dementia. There are 400 MCI subjects with more than one time-point in ADNI database. We selected subjects with 4 time points (i.e. baseline, month 6, month 12 and month 18) FDG-PET data. Patients who converted to AD between baseline and 18 month was excluded, and those who converted to AD during 18 month to 48 month were labeled as pMCI, likewise, the patients whose situation have not changed were labeled as sMCI. Based on the criteria mentioned above, the study population comprised 46 sMCI and 33 pMCI. The demographic and clinical information (MMSE) of all participants at baseline is shown in Table 1. The 46 sMCI patients aged 62–85 at baseline (mean = 77.1; SD = 6.8) (male/female, 31/15), and 33 pMCI patients aged 55–82 at baseline (mean = 73.4; SD = 6.7) (male/female, 24/9). The two groups were relatively well-matched in terms of gender (χ^2^ = 0.2590, *p* = 0.6110). Statistic analysis indicated there were significant differences between sMCI and pMCI patients in age (t = − 2.2933, *p* = 0.0192), and in the demographic variables MMSE (t = − 2.1036, *p* = 0.0387) and ADAS-cog (t = 3.7124, *p* = 3.86e-04) at baseline. We implemented a linear regression to remove the effects of age and gender.

**Table 1:** Subject information

Group	sMCI	pMCI	*p*-value
Number of subjects	46	33	–
Gender(M/F)	31/15	24/9	0.6110^#^
Baseline age (mean ± std)	77.1 ± 6.8	73.4 ± 6.7	0.0192^$*^
Baseline MMSE (mean ± std)	27.8 ± 1.4	27.1 ± 1.6	0.0387^$*^
Baseline ADAS-cog (mean ± std)	13.9 ± 5.5	18.1 ± 4.0	3.86e-04^$*^

*pMCI* progressive mild cognitive impairment, *sMCI* stable mild cognitive impairment, *MMSE* Mini-mental State Examination, *ADAS-cog* Alzheimer’s disease Assessment Scale-Cognitive section. # and $ represent *p*-value for chi-square test and two-sample t-test, respectively. * indicates there are significant differences of the corresponding demographic variables at baseline.

### FDG-PET data acquisition and preprocessing

All FDG-PET Data were acquired using Siemens, GE and Philips PET scanners at resting-state. Details of the PET pre-processing are described at http://adni.loni.usc.edu/methods/pet-analysis/pre-processing/. First, PET images were acquired 30–60 min post-injection at a rate of 1 frame per 5 min. Then, raw PET images were processed to remove the possible differences resulting from scanner differences. For a given subject, each frame was coregistered to the first frame, and then all frames were averaged to generate a single average image. The averaged image was reoriented and filtered into a standard 160 × 160 × 96 voxel image grid with 1.5 mm cubic voxels. The resulting images were smoothed with 8 mm FWHM Gaussian kernels. Finally, all images were spatially normalized to the PET Montreal Neurological Institute (MNI) brain space template, scaled, and averaged using SPM8(Statistical Parametric Mapping 8, http://www.fil.ion.ucl.ac.uk/spm) running under Matlab 7.11(Mathworks Inc., Sherborn, MA, USA) on the CentOS 6.5.

### FDG-PET normalization

Intensity normalization of FDG-PET images is often performed relative to the cerebral global mean. However, subjects with AD have a lower metabolic intensity than MCI across the whole brain [12, 14]. Normalization to the cerebral global mean therefore artificially scales up AD values while scaling down those of MCI cases. Yakushev et al. [36] figured out this problem between AD and normal control. Recent research proposed that using the signal intensity in relatively preserved regions of brain rather than the cerebral global mean value for normalization can improve group discrimination [37]. Yakushev et al. proposed a different approach for defining a reference cluster for normalization. This method consists of 2 steps. First, a cerebral global mean normalization is performed. Then, a two sample t-test is conducted in order to find the apparently hypermetabolic (*p*-value< 0.05) regions in the patient group compared to the healthy control, and these regions are then selected as the reference cluster. In our work, intensity normalization of the FDG-PET images was conducted performing this reference cluster method. The detailed information of the healthy control group was shown in the Supplementary table 1. From our calculation, the location of the reference clusters of different time points mainly included Precuneus, Limbic Lobe, and Posterior Cingulate. Visualization of the reference clusters of different time points was shown in Supplementary Figure 1.

### Feature extraction

After preprocessing and normalization, we extracted the voxels and performed a linear regression to remove the effects of the age and gender. Then voxels were mapping into 246 regions according to the BNA template proposed by the Institute of Automation, Chinese Academy of Sciences. The BNA template is based on standard MNI space, with 210 cortical and 36 subcortical sub-regions, and provides a fine-grained, cross-validated atlas, containing information on both anatomical and functional connections [35]. Average metabolic intensity of regions were taken as static features.

Two types of dynamic features were defined, including intensity differences (D) and the intensity change rate(R). D is the intensity differences between baseline and the other three time points. To obtain *R* values, the D values between baseline and the remaining 3 time points were then divided by the intensity of baseline. The calculation formulas are as follows:

1 $$ {D}_i={T}_{baseline}-{T}_i $$

2 $$ {R}_i=\frac{D_i}{T_{baseline}}\left(i=1,2,3\right) $$

T is the metabolic intensity of each time point. Cognitive features are the MMSE and ADAS-cog scores of 4 time points.

### Feature selection

To increase the classification accuracy, effective feature selection was used for dimensionality reduction, data minimization, redundancy minimization, and calculation reduction. We applied F-score feature selection method, which shows good performance on small samples. F-score selects the most effective features by evaluating the resolving power of the feature samples [38]. Given training samples *x*_*k*_ ∈ *R*^*n*^, k = 1, 2, ⋯, l, and dividing the samples into positive and negative categories, the number of positive samples is *n*_+_ while the number of negative samples is *n*_−_, then the F-score of the i-th feature is defined as follows:

3 $$ F(i)=\frac{{\left(\ {\overline{x}}_i^{\left(+\right)}-{\overline{x}}_i\right)}^2+{\left(\ {\overline{x}}_i^{\left(-\right)}-{\overline{x}}_i\right)}^2}{\frac{1}{n_{+}-1}{\sum}_{k=1}^{n_{+}}{\left(\ {x}_{k,i}^{\left(+\right)}-{{\overline{x}}_i}^{\left(+\right)}\right)}^2+\frac{1}{n_{-}-1}{\sum}_{k=1}^{n_{-}}{\left(\ {x}_{k,i}^{\left(-\right)}-{{\overline{x}}_i}^{\left(-\right)}\right)}^2} $$

${\overline{x}}_i$, ${\overline{x}}_i^{\left(+\right)}$ and ${\overline{x}}_i^{\left(-\right)}$ are the averages of the i-th feature of the whole, positive and negative data sets, respectively. ${\overline{x}}_{k,i}^{+}$ is the i-th feature of the k-th positive instance, and ${\overline{x}}_{k,i}^{-}$ is the i-th feature of the k-th negative instance. The discriminating power of the feature is proportional to the *F* value. Therefore, we can set the threshold value to exclude the features with smaller *F* value, so as to achieve the purpose of feature selection.

In addition, LASSO feature selection method [39] was implemented to further test the stability and effectiveness of the features.

### Classification

Based on the selected features above, the commonly used classifier SVM which is based on structural risk minimization and exploits a margin-based criterion was selected for classification [40, 41]. We applied LIBSVM library [42] on MATLAB, and the RBF kernel was utilized because of its good performance on small sample problems [43]. The RBF kernel is defined as follow:

4 $$ K\left({x}_1,{x}_2\right)=\exp \left(-\frac{{\left\Vert {x}_1-{x}_2\right\Vert}^2}{2{\sigma}^2}\right) $$

Where x_1_ and x_2_ are the two feature vectors, and σ is the width of the Gaussian kernel. To obtain a relative unbiased evaluation of classification performance, we applied the leave-one-out cross-validation strategy with feature selection and classifier training only on training set (see Fig. 1b). Specifically, one subject is first left out as testing set, and the remaining ones are used as training set. The entire process is repeated for each subject. Accuracy, sensitivity, and specificity were determined to evaluate the performance of the proposed classification framework. In addition, the Receiver Operating Characteristic (ROC) curve was used to summarize the classifier performance over a range of trade-offs between true-positive and false-positive error rates [44]. Area Under the ROC Curve (AUC) [45] was used as another measure.

## Results

### Feature selection results

**Fig. 2:**
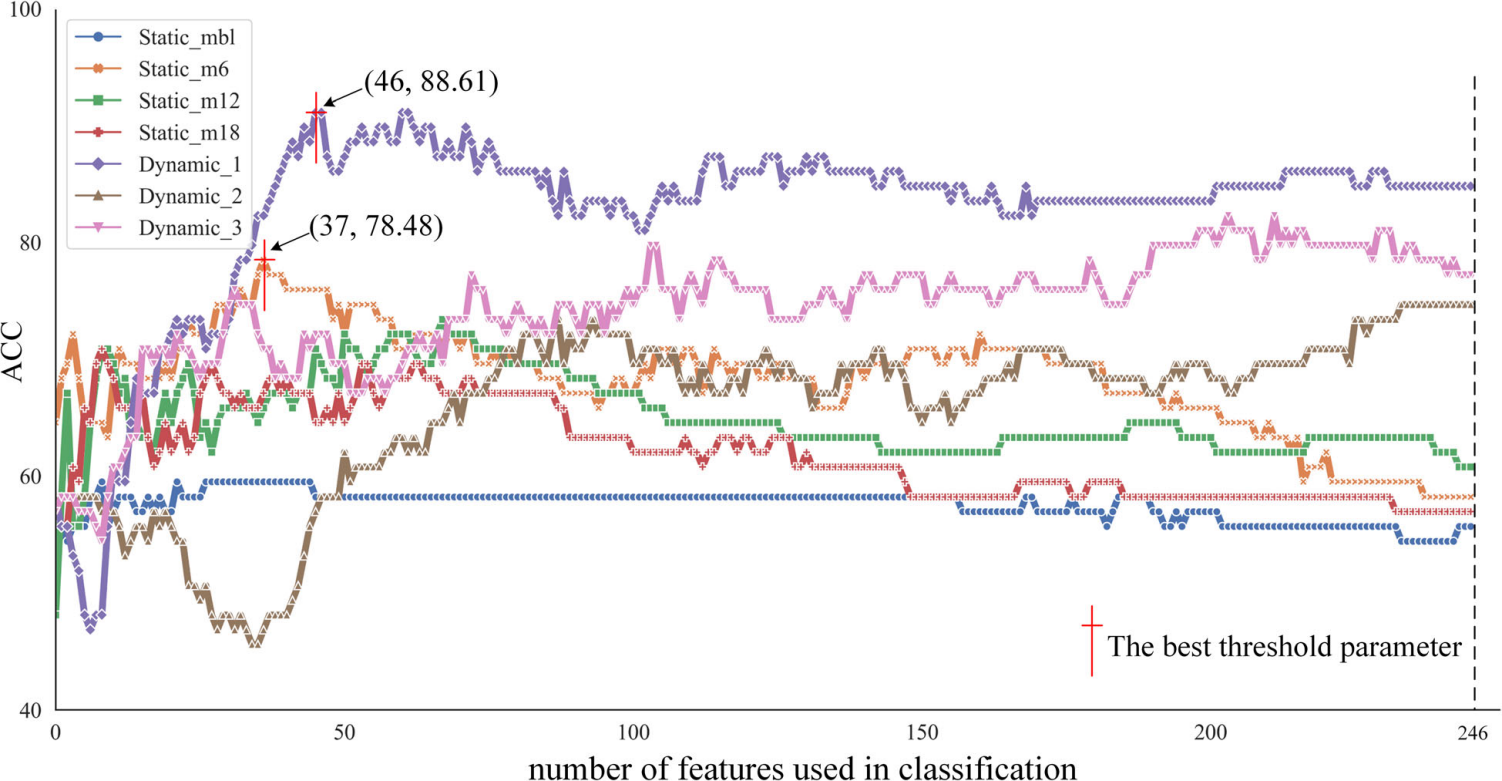
The optimization process of classification. The number of selected features increased from 1 to 246 as shown in x-axis. And the y-axis represents the classification performance with static and dynamic features in different colors and markers. ACC, classification accuracy. Static_mbl, static feature obtained in the baseline. Static_m6, static feature obtained in the 6th month after baseline. Static_m12, static feature obtained in the 12th month after baseline. Static_m18, Static feature obtained in the 18th month after baseline. Dynamic_1, dynamic feature calculated with Static_mbl and Static_m6. Dynamic_2, dynamic feature calculated with Static_mbl and Static_m12. Dynamic_3, dynamic feature calculated with Static_mbl and Static_m18

**Fig. 3:**
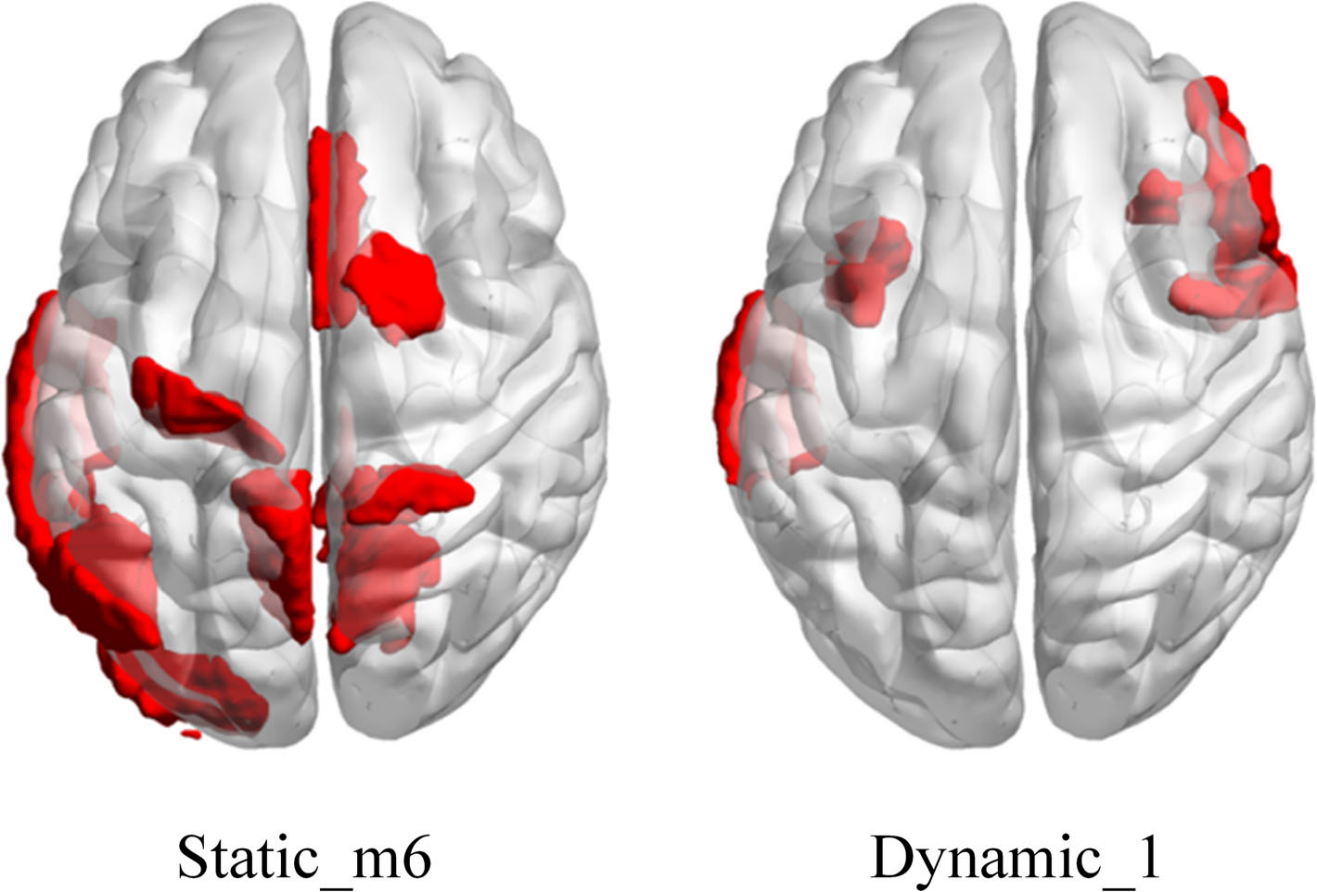
Locations of the selected regions as features overlaid on the standard template. Static_m6, static feature obtained in 6th month after baseline. Dynamic_1, dynamic feature calculated with static feature in baseline and in the 6th month after baseline

By increasing the number of features used in classification, the optimization process of the classification results is shown in Fig. 2. When the number of features was 37, the static features in the 6th month after baseline (Static_m6) obtained the best classification performance among the static features. On the other hand, Dynamic_1 calculated with Static_mbl and Static_m6 obtained the best classification performance among the dynamic features when the number of the selected features equaled to 46. We chose the maximum point of curve of static feature (Static_m6) and dynamic feature (Dynamic_1) respectively. The common features selected from the training set at each leave-one-out were mainly located at some brain regions. We visualized these brain regions in Fig. 3. As for Static_m6, the brain regions included cingulate gyrus (average F-score = 3.85e-5), precuneus (4.49e-5), superior parietal lobule (1.00e-4), superior frontal gyrus (1.27e-4) of right hemisphere and precentral gyrus (5.61e-6), middle temporal gyrus (4.80e-5), inferior temporal gyrus (6.56e-5), precuneus (7.66e-5), inferior parietal lobule (1.59e-4), lateral occipital cortex (1.77e-4) of left hemisphere. As for Dynamic_1, the brain regions mainly included precentral gyrus (3.22e-6), inferior frontal gyrus (5.42e-5), orbital gyrus (1.16e-4) of right hemisphere and middle temporal gyrus (1.19e-5), insular gyrus (1.86e-5) of left hemisphere.

### Classification results

**Fig. 4:**
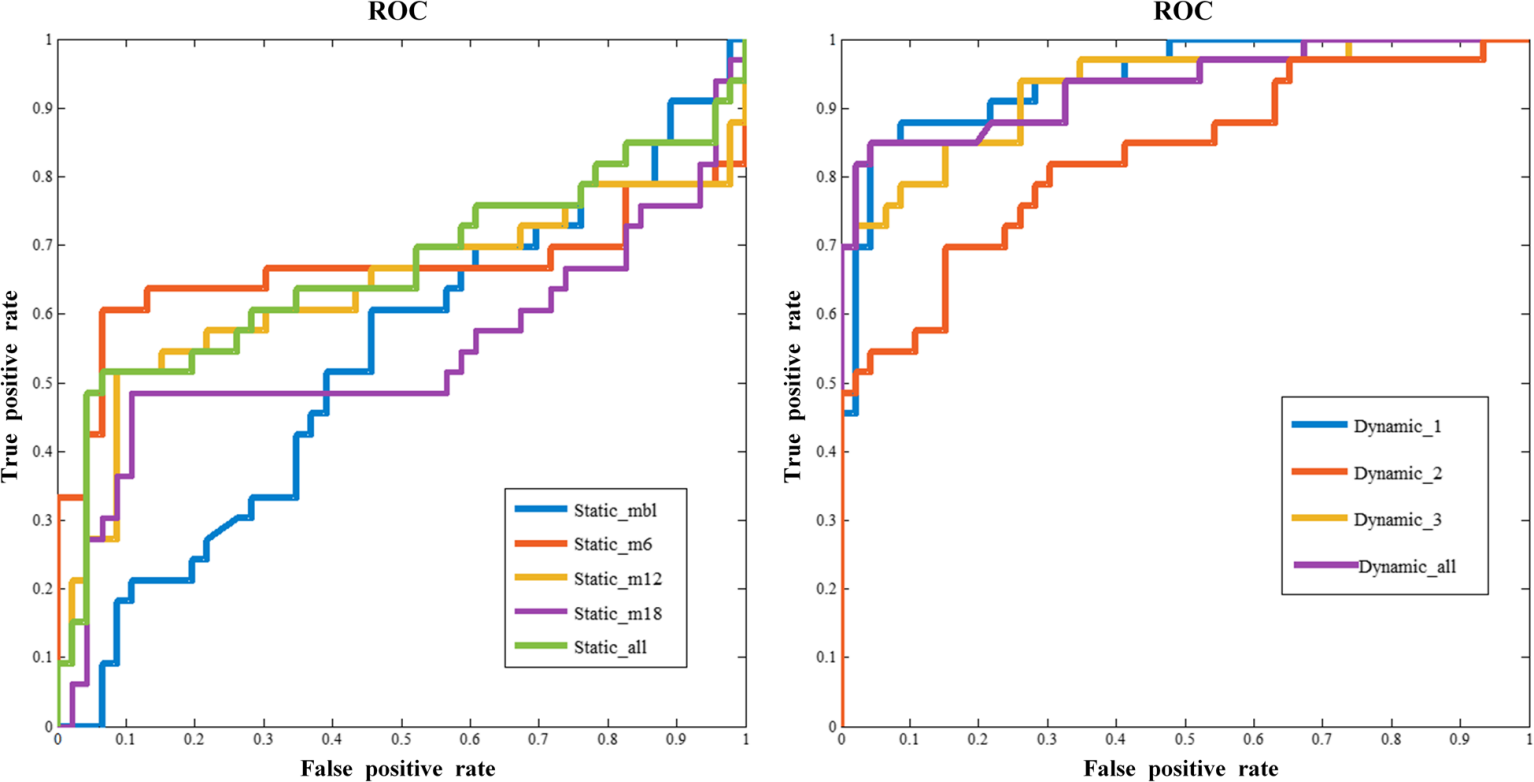
The ROC curves for static features and dynamic features. The ROC curves are at the best performance of classification for static features, dynamic features and the combination of dynamic features and static features respectively

The classification performance was assessed based on three feature sets, which were static, dynamic and cognitive features. As shown in Tables 2 and 3 the performances of average metabolic intensity and metabolic intensity change rate were not satisfactory in classification tasks. Static feature in baseline (Static_mbl) achieved accuracy of 59.49%, sensitivity of 6.06% specificity of 97.83% and AUC of 0.5402. Static features in the 6th month after baseline (Static_m6) achieved accuracy of 78.48%, sensitivity of 57.58%, specificity of 93.48% and AUC of 0.6634. Meanwhile, in the third time point (12th month after baseline, Static_m12), static features achieved accuracy of 73.41%, sensitivity of 48.48%, specificity of 91.30% and AUC of 0.6344. Accuracy of 70.88%, sensitivity of 45.45%, specificity of 89.13%, and AUC of 0.5428 were obtained by implementing static features in the 18th month after baseline (Static_m18). We also combined all static features, but the results did not meet expectations. The combined accuracy was lower than Static_m6 but higher than other three time points. As for dynamic features, the intensity change rates did not shown good classification effect, but the metabolic differences Dynamic_1 calculated by Static_mbl and Static_m6 got the highest accuracy of 88.61% which is better than the static features. All the ROC curves were shown in Fig. 4. In addition, LASSO feature selection based classification results were shown in supplementary table 2 and supplementary table 3. Under LASSO method, Static_m6 obtained the best classification performance with accuracy of 75.94%, sensitivity of 60.61%, specificity of 80.43%, and AUC of 75.96%. As for dynamic features, Dynamic_1 achieved accuracy of 87.34%, sensitivity of 93.94%, specificity of 82.61, and AUC of 0.9302. Compared with LASSO based classification results, F-score based results showed better classification performance both in static features and in dynamic features.

**Table 2:** Comparison of the classification performance in static features

***Feature***	***ACC(%)***	***SEN(%)***	***SPE(%)***	***AUC***
Static_mbl	59.49	6.06	97.83	0.5402
Static_m6	78.48	57.58	93.48	0.6634
Static_m12	73.41	48.48	91.30	0.6344
Static_m18	70.88	45.45	89.13	0.5428
Static_all	75.95	51.52	93.48	0.6614

*ACC* classification accuracy, *SEN* classification sensitivity, *SPE* classification specificity, *AUC* Area Under Curve, *Static_mbl* static feature obtained in the baseline, *Static_m6* static feature obtained in the 6th month after baseline, *Static_m12* static feature obtained in the 12th month after baseline, *Static_m18* static feature obtained in the 18th month after baseline, *Static_all* combining all the static features

**Table 3:** Comparison of the classification performance in dynamic features

***Feature***	***ACC(%)***	***SEN(%)***	***SPE(%)***	***AUC***
**Dynamic_1**	**88.61**	**81.82**	**93.48**	**0.9351**
Dynamic_2	77.21	75.76	78.26	0.8063
Dynamic_3	82.28	73.91	93.94	0.9289
R1	60.76	6.32	100	0.8524
R2	64.56	15.22	100	0.6278
R3	64.56	54.55	71.74	0.5738
Dynamic_all	87.38	87.88	86.96	0.8959

*ACC* classification accuracy, *SEN* classification sensitivity, *SPE* classification specificity. *AUC* Area Under Curve, *Dynamic_1* dynamic feature calculated with Static_mbl and Static_m6 in Table 2, *Dynamic_2* dynamic feature calculated with Static_mbl and Static_m12, *Dynamic_3* dynamic feature calculated with Static_mbl and Static_m18, *R1* metabolic change rate in the 6th month after baseline, *R2* metabolic change rate in the 12th month after baseline, *R3* metabolic change rate in the 18th month after baseline, *Dynamic_all* combining all the dynamic features

Table 4 shows the feature combination results respectively. Combining static features of the 4 time points, an accuracy of 75.95% was obtained which exceeded more than 5% compared with single time point except M6, and the sensitivity and specificity were also improved to 51.52 and 93.38%. Accuracy of combined dynamic features reached 87.38%, higher than that of static features. Cognitive features got a specificity of 93.48%.

**Table 4:** Comparison of the classification performance in the combination features

***Feature***	***ACC(%)***	***SEN(%)***	***SPE(%)***	***AUC***
Static	75.95	51.52	93.48	0.6614
Dynamic	**87.38**	**87.88**	**86.96**	**0.8959**
Cognitive	77.22	55.55	93.48	0.6414
Static & Dynamic	72.15	57.58	82.61	0.7444
Static & Cognitive	73.42	57.58	84.78	0.7345
Dynamic & Cognitive	87.34	75.76	95.65	0.9308
All	72.42	36.36	97.83	0.6443

*ACC* classification accuracy, *SEN* classification sensitivity, *SPE* classification specificity, *AUC* Area Under Curve. Static denotes the combination of all the static features, which is the same as Static_all in Table 2. Dynamic denotes the combination of all the dynamic features as shown in Table 3 (Dynamic_all). Cognitive denotes the combination of all the MMSE and ADAS-cog score features. All denotes the combination of all the types of features

## Discussion

The present study developed a classification framework using both cross-sectional and longitudinal FDG-PET as well as cognitive scores to discriminate pMCI from sMCI. We adequately considered effects from static, dynamic and cognitive features. All these types of features were compared to ensure accurate relationship measurement. Our findings suggested that the dynamic features outperformed previous studies of pMCI and sMCI classification, with an accuracy of 88.61% by SVM RBF [19, 31, 46, 47].

Cerebral glucose metabolism measured by FDG-PET is an impactful mean of MCI prediction. Metabolic intensity reflects integrated synaptic activity. Decreased metabolic intensity in a given brain region indicates either reduced number of synapses or decreased synaptic metabolic activity [48]. Sensitive biomarkers were selected in each type of features, according to Fig. 4, as the middle temporal lobe, cingulate gyrus, inferior frontal gyrus, orbital gyrus, parahippocampal gyrus and postcentral gyrus. In previous studies, many of these selected regions have been indicated as significant in the conversion prediction for MCI patients[49–51].

### Classification performance evaluation

Table 2 demonstrated that combination of all static features yielded an accuracy of 75.95%, achieving better performance in discriminating pMCIs from sMCIs than any single time point except the 6th month after baseline. In this study, result biasing random classification was obtained at baseline. We inferred that the baseline is too far from the disease transformation time point, with little effect on the classification. However, at the early stage of MCI conversion (after baseline), the classification accuracy decreased with time. Therefore, we believed that in the early stage of MCI, the disease deteriorated rapidly. As the disease progresses, the conversion rate slows down. In Table 3, this speculation was further confirmed: Dynamic_1 obtained the highest accuracy and Dynamic_2, Dynamic_3 was lower. As Table 3 shown, Dynamic_all achieved a better prediction accuracy than Dynamic_2, and Dynamic_3 with 10.17 and 5.10% increment respectively. But Dynamic_3 performs better in specificity of 93.94%.

Several studies combined static and dynamic features in MCI classification or prediction [19, 31]. Gray et al. obtained an accuracy of 63.1% while combining longitudinal changes with 12-month FDG-PET signal intensities, with 58.4 and 62.3% for baseline and 12-month signal intensities [31]. Thung et al. combined baseline and 18-month MRI volumetric and dynamic features, and achieved an accuracy of 78.2%, which is 6.6% higher than when using only the reference time point [19]. As shown in Table 4, combination of all the dynamic features achieved the better classification accuracy of 87.38% than the other feature combinations. On the one hand, combining dynamic features and static features did not get a better classification accuracy. But the sensitivity (57.58%) and the AUC (0.7444) was improved compared with the combination of static features (sensitivity of 51.52% and AUC of 0.6614). On the other hand, combining dynamic features and cognitive features achieved better classification performance (accuracy of 87.34%, sensitivity of 75.76%, specificity of 95.65% and AUC of 0.9308) than the combination of cognitive features (accuracy of 77.22%, sensitivity of 55.55%, specificity of 93.48% and AUC of 0.6414). The present results indicated that the dynamic features can provide some complementary information which can enhance classification performance in conjunction with the static features and cognitive features respectively. Additionally, compared with all the static (sensitivity of 51.52% and AUC of 0.6614) and all the cognitive features (sensitivity of 55.55% and AUC of 0.6414), classification performance was improved in sensitivity of 57.58% and AUC of 0.7345 with the combination of static features and cognitive features. This above result showed that static features and cognitive features assistant to each other when predicting pMCIs from sMCIs. When applying all the dynamic, static and cognitive features, classification performance in specificity was improved to 97.83%.

However, it should be noted that the combination of all the features performs worse than some specific combinations. On the one hand, this maybe suffered from the limitations of F-score feature selection method. A disadvantage of F-score is that it does not consider mutual information among features [38]. Despite F-score showed effectiveness on dynamic features, F-score might lack ability in selecting features with complementary information from different feature sets, as static features and cognitive features. On the other hand, the poor performance of the feature combination may due to the over determination or lack of convergence of SVM classifier.

Multiple studies also examined the contribution of cognitive scores [32, 52]. For instance, Cui et al. used different modalities of data, including neuropsychological and functional measures, to explore the optimal set of predictors of conversion from MCI to AD, and obtained an accuracy of 67.13% [52]. Zhang et al. reported that the combination of cognitive scores (MMSE and ADAS-cog) can improve the accuracy, sensitivity, and specificity in distinguishing MCI and AD [32]. In the current study, the combination of MMSE and ADAS-cog scores of 4 time points resulted in lower accuracy of 77.22% and higher specificity of 93.48%. And the combination of cognitive features and static features showed in Table 4 got higher sensitivity of 57.58% and AUC of 0.7345. Also, the combination of cognitive features and dynamic features showed in Table 4 achieved better specificity of 95.65% and AUC of 0.9308.

As shown above, the 88.61% classification accuracy was achieved while using the dynamic features in Dynamic_1 (Table 3). Sensitivity (87.88%, in Table 4) improved by dynamic features and specificity (95.65%, in Table 4) increased by cognitive features strongly contributed to the remarkable results obtained, suggesting that longitudinal data and cognitive scores complementarily enhance the classification performance.

### Methodological limitations

The limitations of this study should be mentioned. First, the combination of multimodal data has been shown to improve the classification results in multiple studies [28, 32, 47, 52, 53]. Other modality data also have different sensitive biomarkers which are complementary in enhancing discrimination performance. In addition, more effective dynamic feature computing methods need to be proposed in the future study.

## Conclusions

Our study suggested that dynamic features got the best classification accuracy than the static features and features of cognitive scores in discriminating pMCIs from sMCIs. And dynamic features and cognitive scores complementarily enhance the classification performance in sensitivity and specificity. Furthermore, the brain regions related to the selected dynamic features might suggest the different progression patterns between pMCIs and sMCIs.

## Supplementary information

**Additional file 1.**

## Data Availability

We obtained permission to access ADNI repository from http://adni.loni.usc.edu/data-samples/access-data/. For readers, login credentials are required to access the ADNI datasets. Readers can easily download the dataset used in this study according to our supplementary file, the Subject-ID.txt once they obtained permission to access ADNI repository. Alternatively, they can contact the corresponding authors by E-mail to get the preprocessed data used in this study.

## Abbreviations

FDG-PET: 18 Fluoro-deoxy-glucose positron emission tomography
AD: Alzheimer’s disease
MCI: Mild cognitive impairment
pMCI: progressive MCI
sMCI: stable MCI
pMCIs: pMCI patients
sMCIs: sMCI patients
ADNI: Alzheimer’s Disease Neuroimaging Initiative
BNA: Brainnetom Atlas
MMSE: Mini-mental State Examination
ADAS-cog: Alzheimer’s disease Assessment Scale-Cognitive section
RBF: Radial bias function
MRI: Magnetic resonance imaging
CFS: Cerebrospinal fluid
SVM: Support vector machine
CDR: Clinical Dementia Rating
MNI: Montreal Neurological Institute
ROC: Receive Operating Characteristic
AUC: Area Under the ROC Curve
