# Supplementary material for: Predicting MCI progression with FDG-PET and cognitive scores: a longitudinal study
Source: BMC Neurol. 2020 Apr 21;20:148. doi: 10.1186/s12883-020-01728-x (PMC7171825; doi:10.1186/s12883-020-01728-x)
Supplement: Supplementary file 1 — Additional file 1. [file 12883_2020_1728_MOESM1_ESM.docx]

# Supplementary Information

1. FDG-PET normalization

Intensity normalization was based on comparing the patient group to a healthy control (HC) group. 75 healthy controls were incorporated into the analysis. Detailed demographic information of the HC group is shown in Table 1.

Table 1 Demographic information of healthy controls

| Group | HC |
| --- | --- |
| Number of subjects | 75 |
| Gender (M/F) | 45/30 |
| Baseline age (mean±std) | 76.26±5.13 |
| Baseline MMSE (mean±std) | 28.91±1.18 |
| Baseline ADAS-cog (mean±std) | 10.47±4.27 |

MMSE, Mini-mental State Examination; ADAS-cog, Alzheimer’s disease Assessment Scale-Cognitive section.

1. The reference cluster results in FDG-PET normalization

In baseline, the reference clusters mainly distributed in Precuneus, Limbic Lobe, Parietal Lobe, Cuneus and Posterior Cingulate. In the second time point, 6^th^ month after baseline, the reference clusters mainly located in Precuneus, Parietal Lobe, Limbic Lobe, Posterior Cingulate, and Occipital Lobe. For the third time point, 12^th^ month after baseline, the reference clusters mainly located in Precuneus, Limbic Lobe, Posterior Cingulate, Occipital Lobe, and Cuneus. In the 18^th^ month after baseline, the third time point, the reference clusters mainly distributed in Precuneus, Limbic Lobe, Parietal Lobe, Posterior Cingulate, Occipital Lobe, and Cingulate Gyrus. Visualization of the reference clusters of difference time points is shown in Figure 1.

**
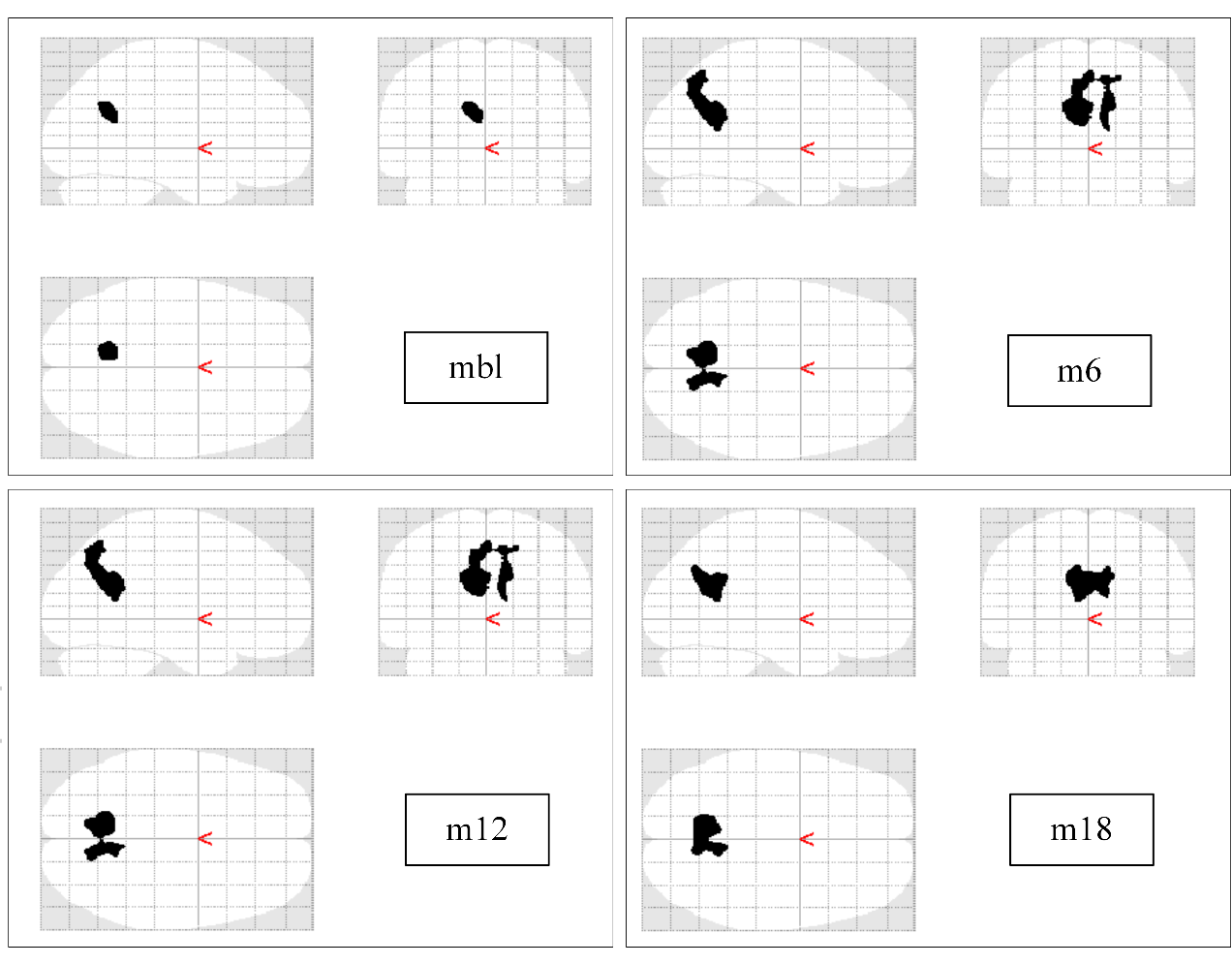
**

**Figure 1. Mask for normalization in different time point of scan.** mbl, month of baseline. m6, 6^th^ month after baseline. m12, 12^th^ month after baseline. m18, 18^th^ month after baseline.

1. **LASSO based classification results**

Table 2. Comparison of the classification performance in static features

| ***Feature*** | ***ACC(%)*** | ***SEN(%)*** | ***SPE(%)*** | ***AUC*** |
| --- | --- | --- | --- | --- |
| Static_mbl | 65.82 | 54.55 | 73.91 | 0.6462 |
| Static_m6 | 70.89 | 57.58 | 80.43 | 0.7358 |
| Static_m12 | 75.94 | 60.61 | 86.96 | 0.7596 |
| Static_m18 | 72.15 | 60.61 | 80.43 | 0.7115 |
| Static_all | 70.89 | 63.64 | 76.09 | 0.6607 |

ACC, classification accuracy. SEN, classification sensitivity. SPE, classification specificity. AUC, Area Under Curve. Static_mbl, static feature obtained in the baseline. Static_m6, static feature obtained in the 6^th^ month after baseline. Static_m12, static feature obtained in the 12^th^ month after baseline. Static_m18, static feature obtained in the 18^th^ month after baseline. Static_all, combining all the static features.

Table 3. Comparison of the classification performance in dynamic features

| ***Feature*** | ***ACC(%)*** | ***SEN(%)*** | ***SPE(%)*** | ***AUC*** |
| --- | --- | --- | --- | --- |
| **Dynamic_1** | **87.34** | **93.94** | **82.61** | **0.9302** |
| Dynamic_2 | 70.89 | 54.55 | 82.61 | 0.7401 |
| Dynamic_3 | 81.01 | 69.70 | 89.13 | 0.8047 |
| R1 | 60.76 | 9.09 | 97.83 | 0.5440 |
| R2 | 64.56 | 21.21 | 97.83 | 0.6173 |
| R3 | 63.29 | 12.12 | 1 | 0.5738 |
| Dynamic_all | 84.81 | 96.97 | 76.09 | 0.9275 |

ACC, classification accuracy. SEN, classification sensitivity. SPE, classification specificity. AUC, Area Under Curve. Dynamic_1, dynamic feature calculated with Static_mbl and Static_m6 in table 2. Dynamic_2, dynamic feature calculated with Static_mbl and Static_m12. Dynamic_3, dynamic feature calculated with Static_mbl and Static_m18. R1, metabolic change rate in the 6^th^ month after baseline. R2, metabolic change rate in the 12^th^ month after baseline; R3, metabolic change rate in the 18^th^ month after baseline. Dynamic_all, combining all the dynamic features.
